# Stress-Induced Proteasome Sub-Cellular Translocation in Cardiomyocytes Causes Altered Intracellular Calcium Handling and Arrhythmias

**DOI:** 10.3390/ijms25094932

**Published:** 2024-04-30

**Authors:** Shunit Neeman-Egozi, Ido Livneh, Irit Dolgopyat, Udi Nussinovitch, Helena Milman, Nadav Cohen, Binyamin Eisen, Aaron Ciechanover, Ofer Binah

**Affiliations:** 1 Department of Physiology, Biophysics and Systems Biology, Rappaport Faculty of Medicine, Technion—Israel Institute of Technology, Haifa 3190601, Israel; shunit36@gmail.com (S.N.-E.); binyae@gmail.com (B.E.); 2 The Rappaport-Technion Integrated Cancer Center (R-TICC) and The Rappaport Faculty of Medicine and Research Institute, Technion-Israel Institute of Technology, Haifa 319060, Israel; idoliv@campus.technion.ac.il (I.L.); 2arnevet@gmail.com (N.C.); 3 Department of Cardiology, Edith Wolfson Medical Center, Holon 5822012, Israel; 4 The Faculty of Medicine, Tel Aviv University, Tel Aviv 6997801, Israel

**Keywords:** ubiquitin–proteasome-system (UPS), proteasome sub-cellular compartmentalization, amino acids starvation, induced pluripotent stem cell-derived cardiomyocytes (iPSC-CMs), intracellular Ca^2+^ handling, arrhythmias

## Abstract

The ubiquitin–proteasome system (UPS) is an essential mechanism responsible for the selective degradation of substrate proteins via their conjugation with ubiquitin. Since cardiomyocytes have very limited self-renewal capacity, as they are prone to protein damage due to constant mechanical and metabolic stress, the UPS has a key role in cardiac physiology and pathophysiology. While altered proteasomal activity contributes to a variety of cardiac pathologies, such as heart failure and ischemia/reperfusion injury (IRI), the environmental cues affecting its activity are still unknown, and they are the focus of this work. Following a recent study by Ciechanover’s group showing that amino acid (AA) starvation in cultured cancer cell lines modulates proteasome intracellular localization and activity, we tested two hypotheses in human induced pluripotent stem cell-derived cardiomyocytes (iPSC-CMs, CMs): (i) AA starvation causes proteasome translocation in CMs, similarly to the observation in cultured cancer cell lines; (ii) manipulation of subcellular proteasomal compartmentalization is associated with electrophysiological abnormalities in the form of arrhythmias, mediated via altered intracellular Ca^2+^ handling. The major findings are: (i) starving CMs to AAs results in proteasome translocation from the nucleus to the cytoplasm, while supplementation with the aromatic amino acids tyrosine (Y), tryptophan (W) and phenylalanine (F) (YWF) inhibits the proteasome recruitment; (ii) AA-deficient treatments cause arrhythmias; (iii) the arrhythmias observed upon nuclear proteasome sequestration(-AA+YWF) are blocked by KB-R7943, an inhibitor of the reverse mode of the sodium–calcium exchanger NCX; (iv) the retrograde perfusion of isolated rat hearts with AA starvation media is associated with arrhythmias. Collectively, our novel findings describe a newly identified mechanism linking the UPS to arrhythmia generation in CMs and whole hearts.

## 1. Introduction 

The ubiquitin–proteasome system (UPS) is one of the two major proteolytic systems in eukaryotic cells. The selective degradation of substrate proteins is facilitated via their conjugation with moieties of ubiquitin, a 9 KDa protein that serves as the recognition signal for protein removal. Ubiquitinated proteins are recognized by the catalytic arm of the UPS —the proteasome, a ~2.5 MDa complex responsible for protein breakdown into short peptides, which are further catalyzed into amino acids (AAs) [1,2]. The UPS was shown to regulate protein degradation in response to different types of stresses [3,4], and serves by itself as a substrate for protein breakdown [5]. More specifically, the UPS was shown to play a key role in cardiac pathophysiology as cardiomyocytes (with very limited self-renewal capacity) are particularly prone to protein damage due to constant mechanical and metabolic stress [6,7,8]. Recent evidence suggests that the UPS, which contributes to the regulation of apoptosis, cell mass and sarcomere quality control [7], is involved in virtually all cellular processes, and its dysfunction mediates some of the mechanisms attributable to the pathogenesis of coronary and non-coronary heart diseases [7,8,9]. Decreased proteasomal activity was reported in animal models and human studies of coronary ischemia/reperfusion injury (IRI) [10,11], heart failure [12,13,14] and cardiomyopathies [15,16]. Various cellular mechanisms, such as the regulation of pro-apoptotic proteins and altered expression of ion channels, were described in the context of the proteasome dysfunction observed in pressure overload-induced heart failure in mice [7,12]. Bahrudin et al. [17] reported that the UPS impairment caused by the E334K mutation in cardiac myosin-binding protein C (cMyBPC) modifies the levels of channel proteins (e.g., K(v)1.5, Na(v)1.5, HCN4, Ca(v)3.2, Ca(v)1.2, SERCA, RyR2, and NCX1), leading to electrophysiological dysfunction, which may partly contribute to the observed clinical arrhythmias in hypertrophic cardiomyopathy (HCM) patients. Finally, several groups reported that the UPS becomes dysfunctional during myocardial ischemia and IRI [10,18,19]. 

The mechanisms responsible for the regulation of UPS activity have been extensively studied in the past few decades, including ubiquitin activation, proteasome assembly, the phosphorylation of both the proteasome and its substrate proteins, the formation, length, and removal of ubiquitin chains by ubiquitinating enzymes (i.e., E3 ubiquitin ligases) and deubiquitinating enzymes (DUBs), and the subcellular compartmentation of the proteasome [20]. A recent study by Livneh et al. [21] showed that in cultured cancer cell lines under stress, the proteasome is translocated from its large nuclear pool to the cytoplasm, where most of its protein substrates reside. This nucleo-cytoplasmic proteasome translocation was shown to serve as an essential stress coping mechanism, facilitating protein breakdown in the cytoplasm.

In the process of dissecting the mechanism governing proteasome translocation in cancer cells, it was found to be regulated by the aromatic amino acids tyrosine (Y), tryptophan (W) and phenylalanine (F) (YWF), which act as agonists of the mTOR signaling pathway [21]. We discovered that the deprivation of all AAs led to proteasome recruitment from the nucleus to the cytoplasm, thereby facilitating cell survival under nutrient shortage. In contrast, administrating YWF to cells lacking all other AAs resulted in proteasome sequestration in the nucleus. Importantly, while stress by itself is tolerable, in part due to proteasome translocation and increased cytoplasmic activity, the inhibition of proteasome recruitment by YWF leads to cancer cell death [21]. 

Whereas many studies have demonstrated the contribution of UPS aberrations to a variety of cardiac pathologies resulting from stress (e.g., IRI), not much is known about the involvement of the proteasome in these disease states, and in particular regarding arrhythmias. Accordingly, in the present work, we tested the following hypotheses in human induced pluripotent stem cell-derived cardiomyocytes—iPSC-CMs (CMs): (i) AA starvation induces proteasome translocation in CMs, similarly to the observation made in cultured cancer cell lines; (ii) the manipulation of subcellular proteasomal compartmentation is associated with electrophysiological abnormalities in the form of arrhythmias, mediated via altered intracellular Ca^2+^ handling. The major findings are: (i) starving CMs to AAs results in proteasome translocation from the nucleus to the cytoplasm, while supplementation with the aromatic YWF inhibits the proteasome recruitment; (ii) AA-deficient treatments cause arrhythmias; (iii) the arrhythmias caused by nuclear proteasome sequestration are blocked by KB-R7943, an inhibitor of the reverse mode of the sodium–calcium exchanger NCX; (iv) the retrograde perfusion of isolated rat hearts with AA-deficient media is associated with arrhythmias. Collectively, our findings describe a newly identified mechanism linking the UPS to arrhythmia generation in iPSC-CMs.

## 2. Results

### 2.1. Starving CMs to Amino Acids Results in Nucleo-Cytoplasmic Proteasome Shuttling, Which Is Inhibited by the Triad of Aromatic Amino Acids

**Figure 1:**
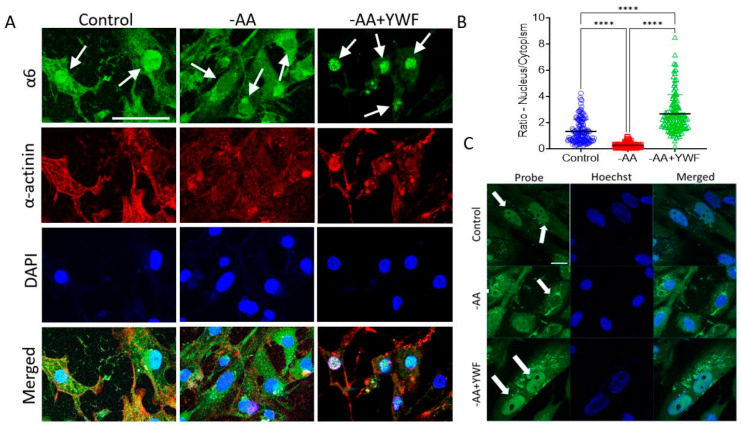
The effect of 2-day amino acid-deficient media on proteasome intracellular distribution in iPSC-CMs (CMs, clone FSE). CMs were cultured in complete medium, control; medium lacking all amino acids, -AA; and -AA medium supplemented with the 3 aromatic amino acids tyrosine (Y), tryptophan (W), and phenylalanine (F), -AA+YWF. (**A**) Representative immunofluorescence images from the three experimental groups; green: staining of the proteasome α6 subunit; red: staining of the cardiac marker α-actinin; blue: DAPI staining of the nucleus. In control the proteasome is mostly concentrated in the nucleus, in -AA the proteasome translocates from the nucleus to the cytoplasm, and in -AA+YWF the proteasome translocation is prevented, and its nuclear signal intensified. Clone: 24.5; scale bar = 50 μm. (**B**) A summary showing the ratio between the proteasome α6 subunit density in the nucleus and cytoplasm, N/C. In each cell, the density was defined as signal intensity divided by the respective areas of the nucleus and cytoplasm. The confocal fluorescent images were analyzed using ImageJ quantification tool (see details in Section 4: Materials and Methods). Statistical analysis was performed by means of the Kolmogorov–Smirnov normality test followed the Kruskal–Wallis test; **** *p* < 0.0001. Control n = 154 CMs; -AA n = 145 CMs; -AA+YWF n = 113 CMs. (**C**) The effect of 2-day amino acid-deficient media on proteasome activity in iPSC-CMs (CMs). Live imaging of proteasome activity was performed using the Me4BodipyFL-Ahx3Leu3VS probe in CMs cultured as in panel A. Green: Me4BodipyFL-Ahx3Leu3VS, a probe for proteasome activity; blue: Hoechst staining the nucleus. The intracellular distribution of proteasome activity is compatible with the α6 subunit distribution. Clone: FSE; scale bar = 20 μm. In all treatments the white arrows point to the nucleus.

Ciechanover and co-workers recently found in various cancer cell lines derived from epithelial tumors that upon AA starvation, the proteasome is translocated from its large nuclear pool to the cytoplasm, where it facilitates protein breakdown. To test whether this stress coping mechanism is also conserved in the heart, we first subjected the CMs to a culture medium lacking all AAs. As was observed in cancer cells, AA starvation induces proteasome translocation from the nucleus to the cytoplasm also in CMs, underscoring the fundamental role of this coping mechanism (Figure 1A). Notably, the addition of YWF to AA-starving CMs inhibited the proteasome recruitment to the cytoplasm, further establishing that the same signal regulates proteasome compartmentalization in different types of tissues (Figure 1A). Proteasome export to the cytoplasm upon AA-starvation, as well as its nuclear sequestration in the presence of YWF, were further established using a probe for active proteasomes [22] (Figure 1C), emphasizing that the shuttling proteasomes are active, thereby supporting a functional proteolytic role for their redistribution to the cytoplasm. To provide a quantitative measure of the nucleus/cytoplasm proteasome distribution, the proteasome fluorescence signal was expressed as “density”—the signal magnitude in the nucleus (N) or cytoplasm (C) divided by their respective areas, resulting in the ratio N/C of proteasome density (Figure 1B). In agreement with the immunofluorescence and activity findings (Figure 1A and 1C), in control (the proteasome is mostly concentrated within the nucleus), the ratio N/C = 1.30. In the absence of all AAs (the proteasome is translocated from the nucleus to the cytoplasm), the N/C = 0.25 (*p* < 0.0001 compared to control), and, in the presence of -AA+YWF (the proteasome signal is augmented in the nucleus), the ratio N/C = 2.66, larger (*p* < 0.0001) than the control (N/C = 1.30).

### 2.2. Amino Acid Starvation and YWF Supplementation Do Not Affect Action Potential Characteristics, and Are Not Toxic to Cardiomyocytes

**Figure 2:**
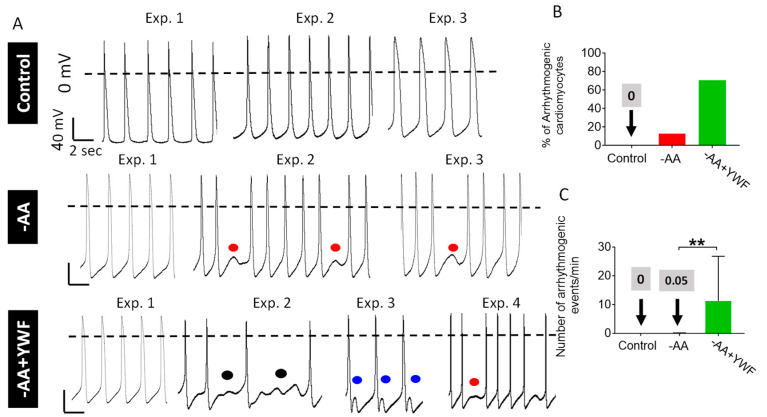
Amino acid-deficient media cause arrhythmias in iPSC-CMs (CMs). (**A**) Representative action potentials recorded from spontaneously firing CMs cultured for 2 days in control (complete medium), -AA and -AA+YWF. The treated CMs contain both regular (left-hand traces) and arrhythmogenic action potentials. Arrhythmias are presented as skipped beats (red symbols), oscillatory pre-potentials (black symbols) and delayed afterdepolarizations (DADs, blue symbols). (**B**) The percentage of arrhythmogenic CMs in control, -AA and -AA+YWF. An arrhythmia is defined as a minimum of three arrhythmogenic events. (**C**) The occurrence of arrhythmias, defined as the mean number of arrhythmogenic events/minute. In the -AA bar, the mean ± SD are 0.0514 ± 0.146. Statistical analysis was performed by means of the Kolmogorov–Smirnov normality test followed by the Mann–Whitney test; ** *p* < 0.01. Control, n = 11 CMs; -AA, n = 9 CMs; -AA+YWF, n = 37 CMs.

**Figure 3:**
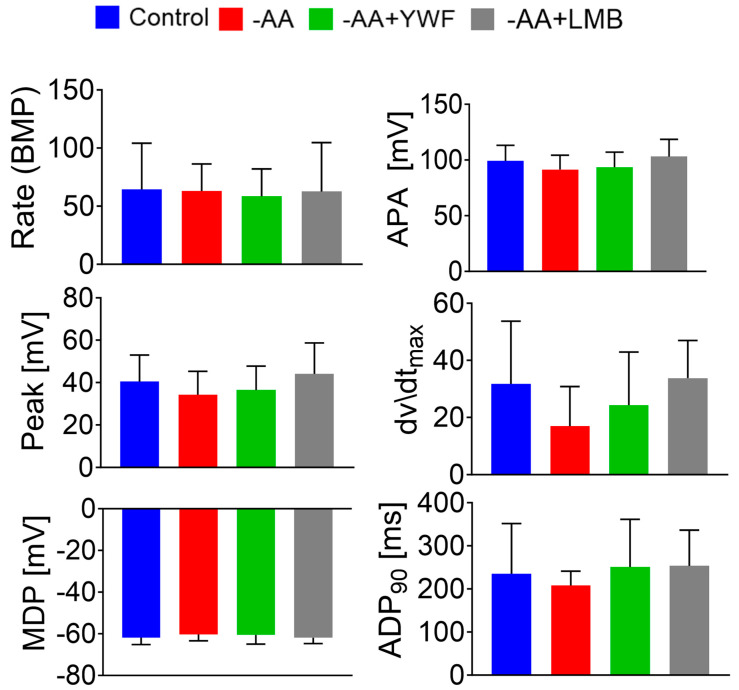
Action potential characteristics recorded from iPSC-CMs (CMs) under different experimental conditions. Control (complete medium): n = 22 CMs; -AA: 2-day treatment, n = 9 CMs; -AA+YWF: 2-day treatment, n = 59 CMs; -AA+Leptomycin B (LMB, 5 ng/mL): 2-day treatment, n = 13 CMs. Beat rate: beats per minute; APA: action potential amplitude; Peak: action potential peak; dV/dt_max_: maximal upstroke velocity of phase zero depolarization; MDP: maximal diastolic potential; APD_90_: action potential duration at 90% repolarization. Statistical analysis was performed by means of the Kolmogorov–Smirnov normality test preformed on all characteristics. Except for dv/dt_max_, all characteristics were normally distributed. For the normally distributed characteristics, we used one-way analysis of variance (ANOVA) followed by Tukey post hoc test. For the non-normally distributed characteristics, we used the Kruskal–Wallis test. There were no statistically significant differences among the four groups.

To determine whether AA starvation and/or YWF administration adversely affect CM function, we recorded and analyzed spontaneously firing action potentials, which constitute a reliable measure of CM viability and functionality. CMs from control, -AA, or -AA+YWF were analyzed for the following characteristics: spontaneous beat rate (beats per minute, BPM), maximal diastolic potential (MDP), action potential amplitude (APA), action potential overshoot (peak), and action potential duration at 90% repolarization (APD_90_). As shown in Figure 2A (left-hand column traces in the three groups) and in Figure 3, all the action potential characteristics in the treated groups are comparable to those of the control group, indicating that neither AA deprivation nor YWF provision are harmful to the electrophysiological function. In summary, in marked contrast to cancer cells in which a brief (4–8 h) exposure to -AA+YWF caused cell death, in CMs, 2-day treatment did not adversely affect the CM function. 

### 2.3. AA-Deficient Treatments Cause Arrhythmias

While AA starvation or YWF supplementation did not affect the action potential characteristics, both treatments caused arrhythmias in otherwise “healthy” CMs (Figure 2). Arrhythmias (more prominent in -AA+YWF than in -AA) were in the form of (i) irregular firing patterns inter-spaced by oscillatory pre-potentials (OPPs, black symbols), (ii) delayed afterdepolarizations (DADs, blue symbols), and (iii) failed beats that appear instead of the regular action potential (red symbols) (Figure 2). Whereas DADs follow regularly generated action potentials, failed beats which have a similar appearance to DADs, result from the failure of action potential generation, and therefore interrupt the regular firing pattern. Further, while DADs appear as a single oscillation or a few damped oscillations, OPPs appear as several oscillations with progressively increasing amplitude. To quantify the arrhythmias, we used two measures: *(1) the percent of arrhythmogenic CMs*, where an arrhythmia is defined as at least three arrhythmogenic events/cell. Whereas the control CMs are arrhythmia-free, the -AA and -AA+YWF-treated CMs presented 12.5% and 70.3% arrhythmogenic CMs, respectively (Figure 2B). *(2) The occurrence of arrhythmias* defined as the mean number of arrhythmogenic events/minute. As depicted in Figure 2C, in -AA+YWF CMs, the occurrence of arrhythmias was higher (*p* < 0.05) than in -AA CMs. 

**Figure 4:**
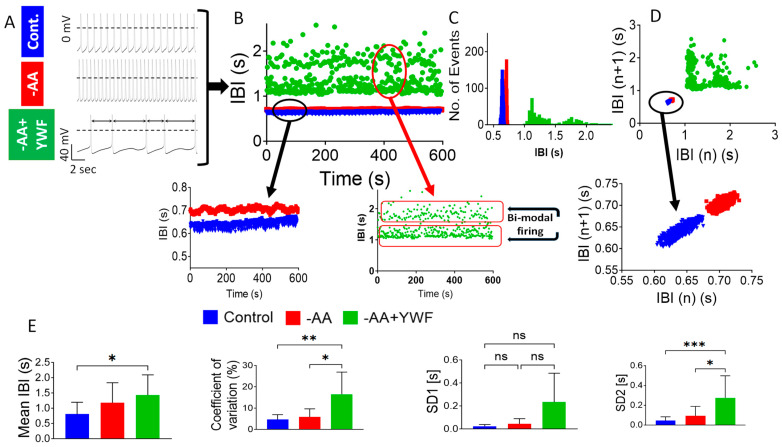
Analysis of Beat Rate Variability (BRV) of spontaneous action potentials recorded from control, and iPSC-CMs (CMs) treated for 2 days with -AA or -AA+YWF. (**A**) Representative action potentials recorded from experiments used for the BRV analysis. (**B**) Inter-beat intervals (IBI) versus time plots of the experiments shown in (**A**). The black arrow in the insert illustrates an expanded IBI scale for control and -AA CMs, showing minimal IBIs dispersion. In contrast to these two groups, -AA+YWF CMs exhibit bimodal firing pattern, as clearly illustrated by the insert, red arrow. (**C**) Superimposed IBI histograms of the three groups. Note the double-peak histogram of the -AA+YWF group. (**D**) Superimposed Poincaré plots of the three groups; whereas the plots of control and -AA are condensed (‘cigar-like’, see insert), the -AA+YWF plot is highly dispersed, composed of more than one cloud. (**E**) BRV measures in control, -AA and -AA+YWF. Mean IBI: mean inter-beat-interval; coefficient of variation; SD1 and SD2: Standard deviation 1 and Standard deviation 2, respectively. Statistical analysis was performed by means of the Kolmogorov–Smirnov normality test followed by the Kruskal–Wallis test; * *p* < 0.05, ** *p* < 0.01, *** *p* < 0.001, ns not significant. Control n = 10 CMs; -AA n = 9 CMs; -AA+YWF n = 17 CMs.

To further quantify the arrhythmias, we analyzed the Beat Rate Variability (BRV), which portrays the dynamic firing patterns of spontaneous action potentials [23,24,25] (Figure 4). Figure 4A depicts the representative action potentials from the experiments used to plot the inter-beat-interval (IBI) versus time in the control, -AA- and -AA+YWF-treated CMs (Figure 4B). Whereas in the control and -AA the IBI versus time plots exhibit small fluctuations around a mean interval (insert, black arrow in panel B), the -AA+YWF CMs exhibit marked IBIs dispersion, as well as a bimodal firing pattern in which the IBIs alternate between two major intervals, with a minimal overlap between the two clouds (Figure 4B, insert, red arrow). This abnormal bimodal firing pattern is represented by a two-peak IBI histogram versus one-peak histogram for the other three groups (Figure 4C). Further, while the Poincaré plot clouds of the control and -AA CMs are condensed (i.e., ‘cigar-like’) (Figure 4D, and insert, black arrow), due to the bimodal firing pattern of the -AA+YWF CMs, the population is much more dispersed and is composed of more than one cloud. In summary, in the control and -AA, the BRV measures coefficient of variation (CV), SD1, and SD2 are comparable. In agreement with the prominent arrhythmias in the -AA+YWF CMs, CV and SD2 were larger than in the -AA and control. Due to the large variability, SD2 was statistically insignificant among the groups.

**Figure 5:**
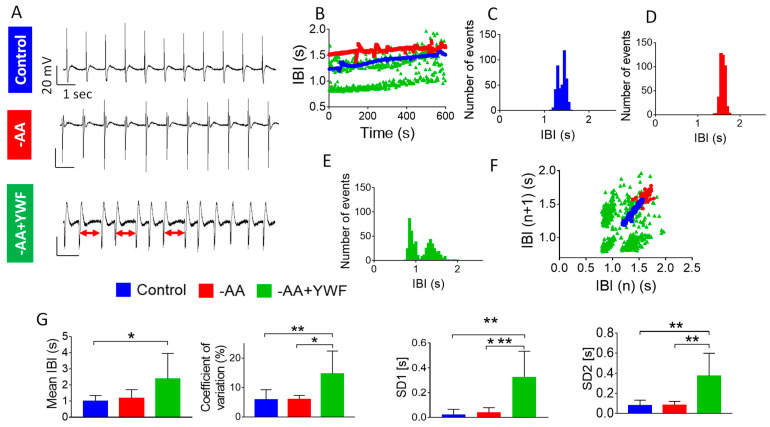
Analysis of Beat Rate Variability (BRV) of extracellular electrograms recorded from control (complete medium) and iPSC-CMs monolayers treated for 2 days with -AA or -AA+YWF. (**A**) Representative extracellular electrograms recorded from spontaneously firing iPSC-CMs clusters used for the BRV analysis. (**B**) Inter-beat intervals (IBI) versus time plots of the experiments shown in (**A**). (**C**–**E**) IBI histograms of control, -AA and -AA+YWF, respectively. Note the double-peak histogram of the -AA+YWF group. (**F**) Superimposed Poincaré plots of the three groups; whereas the plots of control and -AA are condensed (‘cigar-like’), the -AA+YWF plot is highly dispersed, and composed of four clouds as expected from bimodal firing pattern. (**G**) BRV measures in control, -AA, and -AA+YWF. Mean IBI: mean inter-beat-interval; coefficient of variation; SD1 and SD2: Standard deviation 1 and Standard deviation 2, respectively. Statistical analysis was performed by means of the Kolmogorov–Smirnov normality test followed by the Kruskal–Wallis test. * *p* < 0.05, ** *p* < 0.01, *** *p* < 0.001. Control, n = 8 monolayers; -AA, n = 6 monolayers; -AA+YWF, n = 6 monolayers.

To strengthen the single-cell-level findings, we recoded extracellular electrograms from beating CM clusters/networks using the microelectrode array (MEA) setup (Figure 5). Panels A–E depict representative extracellular electrograms (A), IBIs versus time plots (B), and IBIs histograms from the control (C), -AA (D) and -AA+YWF (E) CMs, respectively. Similar to the single-cell findings, at the network level, the -AA+YWF group displayed a bimodal firing pattern (Figure 5B; see two green clouds), two-peak compared to one-peak IBIs histogram plots (Figure 5E) and a Poincaré plot composed of the theoretically expected four discrete clouds (Figure 5F) [24]. Accordingly, in the -AA+YWF group the BRV measures CV, SD1 and SD2 were larger than the control and the -AA group (Figure 5G), once again corroborating the prominent arrhythmogenic nature of the -AA+YWF treatment, at the single-cell level as well as at the network level. 

### 2.4. Supplementing -AA with Leptomycin B (LMB) Causes Proteasome Nuclear Sequestration and Arrhythmias

**Figure 6:**
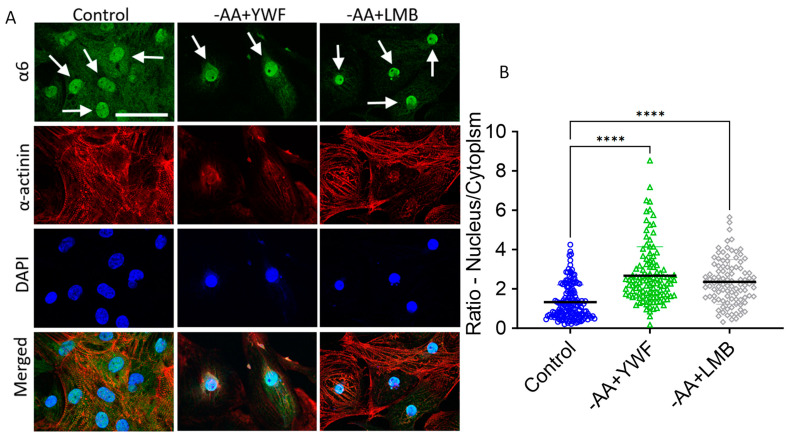
Two-day treatment of iPSC-CMs (CMs) with -AA+Leptomycin B (LMB, 5 ng/mL, an inhibitor of exportin1) recapitulates the effects of -AA+YWF on proteasome intracellular distribution. CMs were cultured for 2 days in control (complete medium), -AA or -AA medium supplemented with LMB (-AA+LMB). (**A**) Representative immunofluorescence images from the three experimental groups; green: staining of the proteasome α6 subunit; red: staining of the cardiac marker α-actinin; blue: DAPI staining of the nucleus. In control, the proteasome is mostly concentrated in the nucleus, whereas in -AA+YWF and -AA+LMB the proteasome nuclear signal is markedly augmented. Clone: FSE; scale bar = 50 μm. The white arrows point to the nucleus. (**B**) A summary showing the ratio between the proteasome α6 subunit density in the nucleus and cytoplasm, N/C. In each cell, the density was defined as signal intensity divided by the respective areas of the nucleus and cytoplasm. The confocal fluorescent images were analyzed using ImageJ quantification tool (see details in Section 4: Materials and Methods). Statistical analysis was performed by means of the Kolmogorov–Smirnov normality test followed by the Kruskal–Wallis test; **** *p* < 0.0001. Control, n = 103 CMs; -AA+YWF, n = 113 CMs; -AA+LMB, n = 101 CMs.

**Figure 7:**
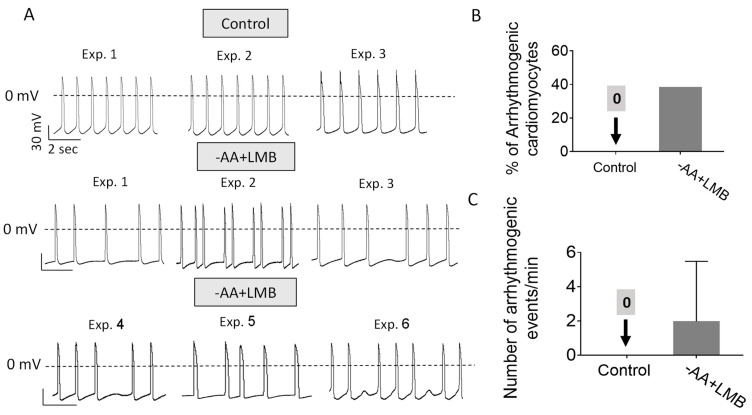
Leptomycin B (LMB, 5 ng/mL) prevents the translocation by -AA of the proteasome from the nucleus to the cytoplasm (-AA+LMB) and causes arrhythmias. (**A**) Representative action potentials recorded from spontaneously firing iPSC-CMs cultured in control (complete medium, 3 experiments) and in -AA+LMB (6 experiments); arrhythmias are shown in all 6 experiments. (**B**) Analysis of the arrhythmias caused by 2-day treatment with -AA+LMB medium versus control. The percentage of arrhythmogenic cardiomyocytes in control and -AA+LMB. (**C**) The occurrence of arrhythmias: the mean number of arrhythmic events/minute in each group. Statistical analysis was performed by means of the Kolmogorov–Smirnov normality test followed by the Mann–Whitney test between the control and -AA+LMB; *p =* 0.0568. Control, n = 8 CMs; -AA+LMB, n = 13 CMs.

To determine whether the translocation of the proteasome to the nucleus is the underlying trigger for the generation of arrhythmias, we tested whether Leptomycin B (LMB), an inhibitor of Exportin1 [26], shown by Livneh et al. [21] to block starvation-induced translocation of the proteasome from the nucleus to the cytosol, will induce arrhythmias, as did the -AA+YWF treatment. Indeed, like -AA+YWF, -AA+LMB causes nuclear sequestration of the proteasome (Figure 6A), resulting in the ratio N/C = 2.36, comparable to -AA+YWF and larger than the control (N/C = 1.30) (*p* < 0.001 compared to control). Notably, AA+LMB treatment was associated with arrhythmias (Figure 7); the percentage of arrhythmogenic CMs was 39% (Figure 7B), and the arrhythmia occurrence in -AA+LMB was 2.0 (Figure 7C). Like YWF (-AA+YWF), while causing arrhythmias, LMB (-AA+LMB) did not affect the action potential characteristics (Figure 3, gray bars). In summary, both -AA+YWF and -AA+LMB cause the translocation of the proteasome from the cytoplasm to the nucleus as well as arrhythmias.

### 2.5. Do AA-Deficient Treatments Affect Intracellular Ca^2+^ Handling?

Many studies showed that a variety of arrhythmias including DADs [27,28,29,30] and OPPs [31,32] are initiated by elevated intracellular Ca^2+^ concentration (i.e., [Ca^2+^]_i_-overload). Accordingly, we employed three protocols aimed to determine whether the AA-deficient treatments (causing arrhythmias) affect intracellular Ca^2+^ handling, which results in elevated [Ca^2+^]_i_: (i) measuring Ca^2+^ transients and caffeine-induced RyR-mediated sarcoplasmic reticulum (SR) Ca^2+^ release; (ii) testing the ability of rapid pacing to cause arrhythmias; and (iii) testing the involvement of NCX in the arrhythmias caused by -AA+YWF.

(i) Measuring Ca^2+^ transients and caffeine-induced RyR-mediated SR Ca^2+^ release

**Figure 8:**
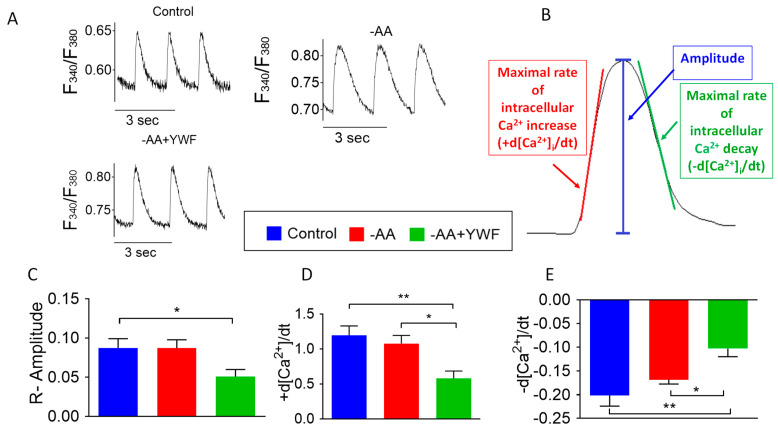
Ca^2+^ transients in control (complete medium) and in iPSC-CMs (CMs) treated for 2 days with -AA or -AA +YWF. (**A**) Representative Ca^2+^ transients from the three experimental groups. (**B**) A scheme illustrating the Ca^2+^ transient characteristics analyzed. (**C**) Ca^2+^ transient amplitude (R-Amplitude). (**D**) Maximal rate of [Ca^2+^]_i_ rise (+d[Ca^2+^]_i_ /dt). (**E**) Maximal rate [Ca^2+^]_i_ decay (−d[Ca^2+^]_i_ /dt. Statistical analysis was performed by means of the Kolmogorov–Smirnov normality test, followed by one-way analysis of variance (ANOVA) and Tukey post hoc test; * *p* < 0.05, ** *p* < 0.01. Control, n = 8 clusters; -AA, n = 7 clusters; -AA+YWF, n = 9 clusters.

To test whether the arrhythmia-causing treatments affect intracellular Ca^2+^ handling, we recorded Ca^2+^ transients from the control, -AA- and -AA+YWF-treated CMs (Figure 8A). Following the analysis depicted in Figure 8B, we found that in the -AA+YWF CMs: (i) the Ca^2+^ transient amplitude is smaller than in the control (Figure 8C); (ii) the maximal rate of Ca^2+^ rise (+d[Ca^2+^]_i_/dt) and the maximal rate of Ca^2+^ decay (−d[Ca^2+^]_i_/dt) are smaller than in the control and -AA (Figure 8D and Figure 8E, respectively). 

**Figure 9:**
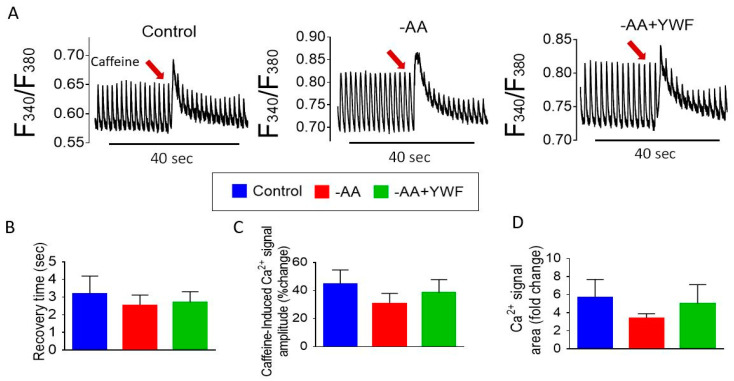
The response of the Ca^2+^ transient to caffeine in control (complete medium) and in iPSC-CMs treated for 2 days with -AA or -AA+YWF. (**A**) Representative Ca^2+^ transients illustrating the response to caffeine in the three experimental groups. The red arrows indicate the addition of caffeine. (**B**) Recovery time calculated as the time from the peak of caffeine-induced Ca^2+^ rise to the first measurable Ca^2+^ transient. (**C**,**D**) The percent change in caffeine-induced Ca^2+^ transient amplitude, and fold change in the area of caffeine-induced Ca^2+^ transient compared to the pre-caffeine Ca^2+^ transient, respectively. The statistical analysis was performed by means of the Kolmogorov–Smirnov normality test, followed by one-way analysis of variance (ANOVA) and Tukey post hoc test. There were no statistically significant differences among the three groups. Control, n = 8 clusters; -AA, n = 7 clusters; -AA+YWF, n = 9 clusters.

Another key feature of the Ca^2+^ handling machinery is the ability of the sarcoplasmic reticulum (SR) to release Ca^2+^ in response to a brief caffeine (a RyR opener) application (Figure 9) [33,34]. In agreement with our previous reports [34,35], caffeine caused an abrupt increase in the Ca^2+^ transient amplitude, followed by a decline in the intracellular Ca^2+^ levels along with the resumption of the transients within several seconds (Figure 9A) [34,36]. To quantify the response to caffeine, we calculated three parameters: (i) the mean recovery time—the time from the peak of caffeine-induced intracellular Ca^2+^ rise to the first measurable Ca^2+^ transient; (ii) the percent change in caffeine-induced Ca^2+^ transient amplitude, compared to the pre-caffeine amplitude; and (iii) the fold change in the area of the caffeine-induced Ca^2+^ transient, compared to the pre-caffeine Ca^2+^ transient area. The summary of these experiments shows (Figure 9B–D) that caffeine caused a similar effect in the three groups, demonstrating that the AA-deficient treatments were not harmful to the CMs. The findings presented in Figure 8 and Figure 9 suggest that: (i) the decline in the Ca^2+^ transient characteristics in the -AA+YWF group could result from smaller SR Ca^2+^ stores; (ii) the caffeine-induced Ca^2+^ release machinery is intact in the -AA and -AA+YWF groups.

(ii) Testing the ability of rapid pacing to cause arrhythmias

**Figure 10:**
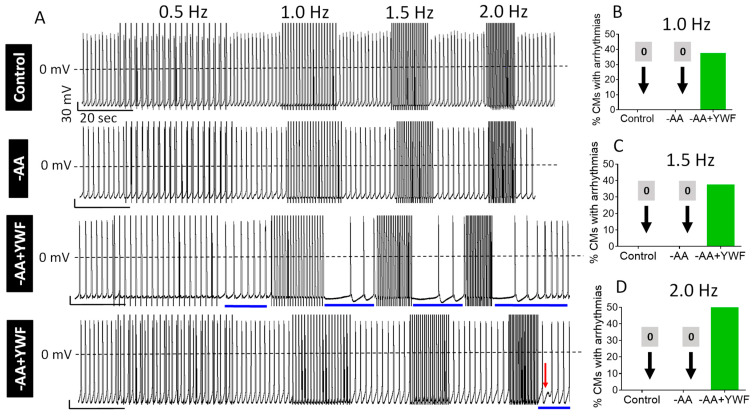
Rapid pacing causes arrhythmias in -AA+YWF (2-day treatment) but not in control or -AA (2-day treatment) iPSC-CMs (CMs). The pacing protocol consists of 20-pulse trains at 0.5, 1.0, 1.5, and 2.0 Hz, with a 20 s pause interval between each pacing session. (**A**) Representative action potentials recordings of the pacing protocols, illustrating that in contrast to control (upper row) and -AA (2^nd^ row from top), rapid pacing induced arrhythmias in -AA+YWF (3^nd^ and 4^rd^ rows from top). The red arrow points to a delayed afterdepolarization. (**B**–**D**) Percentage of CMs presenting arrhythmias in response to pacing at 1.0 Hz, 1.5 Hz, and 2.0 Hz. Compared to control and -AA, 37–50% of -AA+YWF CMs generated arrhythmias in response to rapid pacing. Control, n = 6 CMs; -AA, n = 5 CMs; -AA+YWF, n = 8 CMs.

Because a variety of arrhythmias and DADs in particular, are initiated by elevated intracellular Ca^2+^, we tested whether in the non-arrhythmogenic, regularly firing -AA- and -AA+YWF-treated CMs, arrhythmias will be induced by rapid pacing, a condition causing diastolic Ca^2+^ rise [37]. The pacing protocols consist of 20-pulse trains (pulses delivered at 0.5, 1.0, 1.5, and 2.0 Hz) separated by a 20 s pause (Figure 10). Whereas in the control and -AA CMs rapid pacing did not affect the regular firing patterns, in -AA+YWF pacing caused arrhythmias (marked by the horizontal blue bars), including DADs (shown by the red arrow) and cessation of firing during the post-pacing period (Figure 10A). Notably, at 1.0, 1.5, and 2.0 Hz pacing frequencies, the arrhythmia incidence in -AA+YWF CMs was 37.5%, 37.5%, and 50%, respectively (Figure 10B–D).

(iii) Testing the involvement of NCX in the arrhythmias caused by -AA+YWF

**Figure 11:**
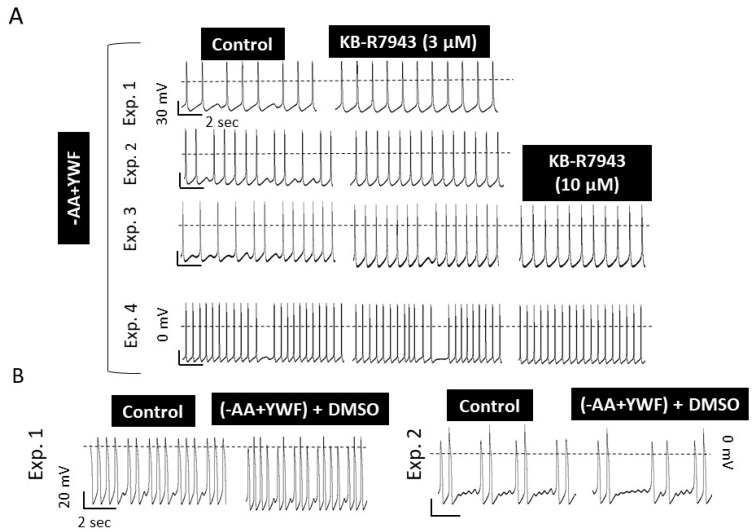
KB-R7943 (a blocker of NCX reverse mode) blocks the arrhythmias in iPSC-CMs (CMs) treated for 2 days with -AA+YWF. (**A**) Four representative experiments show that KB-R7943 (after 4–5 min superfusion) blocked the arrhythmias either at 3 μM or 10 μM. Collectively, KB-R7943 blocked the -AA+YWF-induced arrhythmias in 11 out of 12 experiments. (**B**) CMs treated for two days with -AA+YWF were superfused with Tyrode`s solution, followed by Tyrode’s solution containing 15 µL of dimethyl sulfoxide (DMSO, after 4–5 min superfusion)—the KB-R7943 solvent.

**Figure 12:**
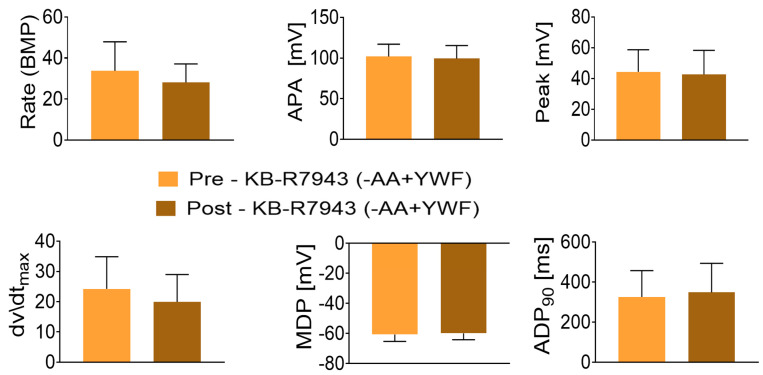
KB-R7943 (a blocker of the NCX reverse mode) does not affect action potential characteristics of 2-day treated -AA+YWF iPSC-CMs (CMs). Action potential characteristics were measured pre-KB-R7943 and post-KB-R7943 (after 4–5 min of superfusion), when the arrhythmias were blocked. Beat rate: beats per minute; APA: action potential amplitude; Peak: action potential peak; dV/dt_max_: maximal upstroke velocity of phase zero depolarization; MDP: maximal diastolic potential; APD_90_: action potential duration at 90% repolarization. The statistical analysis was performed on all characteristics by means of the Kolmogorov–Smirnov normality tests. Except for dv/dt_max_, all characteristics were normally distributed. For the normally distributed characteristics, we used *t*-test. For the non-normally distributed characteristics, we used the Mann–Whitney test. There were no statistically significant differences between the two groups. n = 10 CMs in each group.

The NCX is a transmembrane protein responsible for extruding ~93% of the Ca^2+^ ions that enter the cell during the plateau phase of the action potential, thereby being responsible for the maintenance of Na^+^ and Ca^2+^ homeostasis in cardiomyocytes [38,39]. As DADs and OPPs are known to result from increased cytosolic Ca^2+^ [31,32], we hypothesized that inhibiting by KB-R7943 the reverse-mode of NCX which leads to Ca^2+^ influx into the cell, will alleviate cytosolic Ca^2+^ overload and eliminate the arrhythmias caused by -AA+YWF treatment. In support of this hypothesis, the application of KB-R7943 at 3 or 10 μM blocked the arrhythmias (Figure 11). In summary, in 11 out of the 12 experiments, 3 or 10 μM KB-R7943 blocked the arrhythmias (Figure 11A), suggesting that the inward Ca^2+^ influx via the NCX reverse mode mediates/contributes the arrhythmias caused by -AA+YWF treatment. To test whether, aside from blocking the arrhythmias, KB-R7943 affects CMs’ electrophysiological function, action potentials were analyzed in the CMs in which the arrhythmias were blocked by KB-R7943. As shown in Figure 12, KB-R7943 did not affect the action potential characteristics, suggesting that the anti-arrhythmic effect was specific rather than due to a general attenuation of the electrophysiological function. To negate the possibility that the anti-arrhythmic effect was caused by DMSO—the KB-R7943 solvent, we tested whether DMSO (at the same concentration used to dissolve KB-R7943) alone affects the arrhythmias in -AA+YWF CMs. Figure 11B depicts in two representative experiments (of the three performed), that DMSO does not affect the arrhythmias induced by the -AA+YWF treatment.

### 2.6. Retrograde Perfusion of Isolated Rat Hearts with AA-Deficient Media

**Figure 13:**
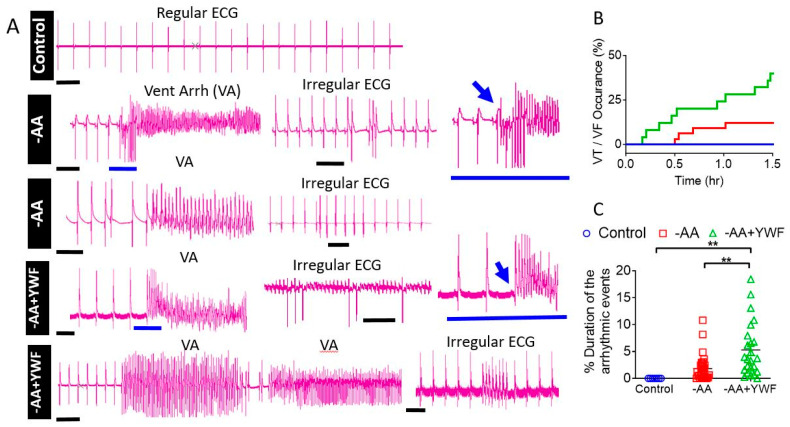
AA-deficient solutions cause arrhythmias in Langendorff retrogradely perfused isolated rat hearts for 1.5 h. Hearts were perfused with Tyrode’s solution with either all amino acids (control), no amino acids (-AA), or only the 3 aromatic amino acids tyrosine (Y), tryptophan (W), and phenylalanine (F) (-AA+YWF). (**A**) Representative ECG recordings illustrating regular firing in a control heart, and ventricular arrhythmias (Vent Arr, VA) in -AA and -AA+YWF perfused hearts. The horizontal blue lines in the main figure and inserts show the abrupt transition (marked by the blow arrows) from a regular ECG to VA bursts. Black scale bar = 0.5 s. Rhythm irregularities were associated mostly with ectopic ventricular rhythms, but occasionally from supraventricular arrhythmias and irregularities in AV conduction. (**B**,**C**) Analysis of the malignant VA (VT and VF) in isolated rat hearts perfused (1.5 h) with control solution, -AA or -AA+YWF. (**B**) The VA occurrence in the perfused hearts. Whereas the control hearts were non-arrhythmogenic, the arrhythmias caused by -AA+YWF were more pronounced than by -AA. (**C**) The fractional (percentage) duration of the arrhythmias during the 1.5 h experiment. The statistical analysis was performed by means of the Kolmogorov–Smirnov normality test, followed by the Kruskal–Wallis test; ** *p* < 0.01. Control, n = 7 rats; -AA, n = 33 rats; -AA+YWF, n = 25 rats.

To extend the implication of the single-cell and network findings to the organ level, isolated rat hearts were retrogradely perfused for 1.5 h with the control, -AA, and -AA+YWF solutions. The study included 7 control (containing all AAs) rats, 33 -AA rats and 25 -AA+YWF rats. As illustrated by the representative ECG recordings, in the control perfusate, the ECG presents a regular beating pattern (Figure 13A). In contrast, the -AA and -AA+YWF perfusates were associated with two types of arrhythmias: (i) irregular ECG patterns, expressed as variable R–R intervals; and (ii) ventricular arrhythmias (Vent Arr, VA), including ventricular tachycardia (VT) and ventricular fibrillation (VF), which erupt abruptly, as illustrated by the blue arrows in the two inserts. In summary, whereas all the control hearts were arrhythmia-free, VA developed in 11.8% of the -AA hearts and 40% in the -AA+YWF (Figure 13B). In agreement with the differences in the occurrence of the VA events between -AA and -AA+YWF hearts, the duration of the arrhythmic events was longer (*p* < 0.001) in -AA+YWF than in -AA (Figure 13C). These findings are of prominent importance because they demonstrate that AA-deficient media cause arrhythmias at the single-cell, network and organ levels, as well as that the arrhythmias caused by -AA+YWF are more pronounced than those caused by -AA.

## 3. Discussion

In the present study, we tested two hypotheses: (i) AA starvation induces proteasome translocation in CMs, similarly to the observation made in cultured cancer cell lines; (ii) the manipulation of subcellular proteasomal compartmentation is associated with electrophysiological abnormalities in the form of arrhythmias, mediated via altered intracellular Ca^2+^ handling. Our main findings are: (i) starving CMs to AAs results in proteasome translocation from the nucleus to the cytoplasm, while YWF supplementation inhibits the proteasome recruitment; (ii) interfering with the stress-induced proteasome translocation in CMs results in prominent arrhythmias; (iii) the arrhythmias observed in the CMs upon nuclear proteasome sequestration are blocked by KB-R7943, an inhibitor of the reverse mode of the NCX; and (iv) the retrograde perfusion of isolated rat hearts with AA starvation media is associated with arrhythmias.

### 3.1. AA-Deficient Media Cause Nucleo-Cytoplasmicc Proteasome Translocation

In general agreement with Livneh et al. [21], exposure of CMs to AA-deficient medium causes proteasome translocation from the nucleus to the cytoplasm, resulting in a smaller N/C ratio (0.25) compared to the control (N/C = 1.30). Supplementing the -AA medium with YWF prevented the proteasome translocation to the cytoplasm, resulting in an N/C ratio of 2.66. Because the YWF intensified the nuclear proteasome signal compared to the control, it is plausible that the YWF not only blocked the -AA-induced proteasome migration to the cytoplasm but also recruited the proteasome from the cytoplasm to the nucleus. In the cytoplasm the UPS is involved in the protein degradation required to replenish the depleted AAs needed for the de novo synthesis of essential proteins and provides the starved cells AAs for energy production [40,41]. Accordingly, in the absence of AAs, the proteasome is recruited from the nucleus to provide the much-needed AAs for protein synthesis. Hence, the proteasome continuously shuttles between the nucleus and the cytoplasm as part of the normal cell response to changing physiological conditions. Intriguingly, despite the common phenomenon (proteasome translocation) and the governing metabolic cue (YWF), the time constants for proteasome translocation differ significantly between cancer cells and CMs. It was shown in cancer cells that the majority of the nuclear proteasome population is shuttling to the cytoplasm following only 4–8 h of starvation [21]. We now show that in CMs the process is much slower—requiring starvation to extend for days to stimulate the proteasome export (Figure 1). We hypothesize that this difference in the kinetics of the proteasome translocation among diverse cell types may represent different susceptibility to stress conditions, as well as dependence on proteasome recruitment in order to cope with AA deprivation. Cancer cells are highly metabolic and rapidly dividing, and while inherently stressed, they are also poorly perfused. Therefore, their requirement for proteasome recruitment is almost immediate, and the consequence of nuclear sequestration is deleterious. In contrast, CMs present proteasome export only after a much-prolonged shortage, reflecting their lesser and partial dependence on this coping mechanism. Accordingly, the lack of any cytotoxic effect following YWF administration to CMs is not surprising. Further, AAs are essential nutrition and energy sources for cancer cell growth and, as intermediates, regulate glucose, lipid and nucleotide metabolism. Hence, cancer cells are dependent on AAs more than healthy cells [42,43,44].

### 3.2. The Effects of AA-Deficient Media on the Electrophysiological Characteristics of CMs

#### 3.2.1. AA-Deficient Media Do Not Affect Action Potential Characteristics

As demonstrated in Figure 3, the AA-deficient treatment (i.e., both -AA and -AA+YWF) did not affect the action potential characteristics. Since overall these characteristics and especially maximal diastolic depolarization (MDP) are affected by the ATP-dependent Na/K-ATPase activity, and are therefore sensitive to the CM bioenergetic and metabolic status, we concluded that these treatments did not adversely affect cell viability.

#### 3.2.2. AA-Deficient Media Cause Arrhythmias in CMs and in Isolated Rat Hearts

AA-deficient treatments are associated with arrhythmias in healthy CMs, as indicated by their control-comparable action potential characteristics (Figure 3). These findings suggest that the arrhythmias were caused by a specific mechanism(s) (discussed below) and not due to cell deterioration, which will adversely affect the action potential characteristics. As indicated by the two arrhythmia measures—the number of arrhythmogenic CMs (Figure 2B) and the number of arrhythmogenic events/min (arrhythmia occurrence; Figure 2C), the arrhythmias caused by -AA+YWF were more pronounced than by -AA. In support of the arrhythmias observed at the single-cell level, the -AA+YWF treatment also cause arrhythmias at the network level (Figure 4), represented by the extracellular electrograms recorded from CM clusters. Respecting the BRV analysis at the single-cell (Figure 4) and network levels (Figure 5), the BRV measures coefficient of variation, SD1 (significant only in the MEA experiments) and SD2 are larger in the -AA+YWF group than in the control and -AA groups, corroborating the arrhythmogenic nature of the -AA+YWF treatment. The prominent arrhythmogenic behavior of the -AA+YWF CMs is demonstrated by the bimodal firing pattern (absent in -AA CMs) at the cell and network levels, as illustrated by (i) the alternating IBIs between the two populations, with minimal overlap (Figure 4B and Figure 5B); (ii) the double-pick IBI histogram (Figure 4C and Figure 5E); and (iii) the markedly dispersed/diffused Poincaré clouds in -AA+YWF, in contrast to a single cloud in the control and -AA groups (Figure 4D and Figure 5F). This bimodality is similar to the bimodal firing pattern caused by GCP-37157 (mitochondrial NCX exchanger antagonist [45]) and ryanodine (sarcoplasmic reticulum [SR] Ca^2+^-induced Ca^2+^-release channel blocker [46]) we reported in iPSC-CMs [24]. A support for the pathological nature of the bimodal firing pattern emerges from the analysis of the Heart Rate Variability (HRV) properties in healthy volunteers and in patients with sick sinus syndrome (SSS) )discussed in reference [24]). In agreement with our findings, the authors’ Figure 3 shows a bimodal heart rate firing pattern observed in (i) the R–R interval versus time plot demonstrating two discrete R–R intervals at 1.0 and 1.6 s, and (ii) the Poincaré plots of patients with SSS depicting four populations. As we cited in our Ben-Ari et al. (2014) paper, “While we cannot offer a mechanistic explanation for the bimodality recorded in drug-treated iPSC-CMs, it is intriguing that the irregular firing pattern recorded in situ in patients with SSS is paralleled by the in vitro firing behavior in iPSC-CMs with disturbed Ca^2+^ handling”. Hence, this bimodality is a strong support for a disturbed Ca^2+^ handling caused by -AA+YWF media. 

The significance of the arrhythmias caused by AA-deficient media at the cell and network level is augmented by the occurrence of arrhythmias in isolated perfused rat hearts (Figure 13). Although the experiments in CMs and isolated rat hearts predominantly differ (i.e., cells versus whole hearts and length and mode of exposure to AA-deficient media), AA and -AA+YWF prominent arrhythmias were induced in both models. 

### 3.3. The Mechanism Underlying the Arrhythmias in -AA+YWF-Treated CMs

The search for the mechanisms underlying the arrhythmias caused by -AA+YWF relied on numerous studies showing that a variety of arrhythmias result from Ca^2+^ overload, as demonstrated by the following examples: (i) DADs are generated by Ca^2+^ overload due to the transient inward current (I_T_i) carried by NCX [47,48]. (ii) Catanzaro and co-workers reported that in a guinea pig sinoatrial node (SAN), the oscillatory afterpotentials and prepotentials are related to a diastolic release of Ca^2+^ from a Ca^2+^-overloaded SR [32]. (iii) We and others [36,49,50,51,52] reported that the DADs in iPSC-CMs derived from Catecholaminergic Polymorphic Ventricular Tachycardiac (CPVT) patients are caused by intracellular Ca^2+^ overload. Accordingly, our efforts were directed towards deciphering whether the -AA+YWF treatment is associated with disturbed Ca^2+^ handling, by means of three protocols.

- (i) Measuring Ca^2+^ transients

We found that the Ca^2+^ transient amplitude, the maximal rates of Ca^2+^ rise (+d[Ca^2+^]_i_/dt) and decay (−d[Ca^2+^]_i_/dt) are smaller in the -AA+YWF than in the control and -AA groups (compared to -AA, smaller for the latter two characteristics) (Figure 8). The decline in the Ca^2+^ transient characteristics in the -AA+YWF group could result from smaller SR Ca^2+^ stores, although this was not supported by a comparable effect of caffeine in the three experimental groups (Figure 9). Smaller SR Ca^2+^ stores can be caused by SR Ca^2+^ leak, which will lead to Ca^2+^ overload, thus giving rise to arrhythmias.

- (ii) Testing the ability of rapid pacing to cause arrhythmias

Rapid pacing can cause arrhythmias in CMs in which diastolic Ca^2+^ is already elevated, yet does not reach the threshold for initiating arrhythmias. In such a circumstance, rapid pacing will further increase intracellular Ca^2+^, thereby exceeding the threshold for triggering arrhythmias. Indeed, in support of a background intracellular Ca^2+^-overload, in contrast to the control and -AA groups, in the -AA+YWF group, 1.0, 1.5, and 2.0 Hz pacing trains caused arrhythmias in 37.5%, 37.5%, and 50% of the CMs, respectively (Figure 10).

- (iii) Testing the involvement of NCX in the arrhythmias caused by -AA+YWF

A remarkable finding was that KB-R7943, which blocks the Ca^2+^ influx generated by the reverse NCX mode [53], blocked the arrhythmias caused by -AA+YWF (Figure 11). This novel finding is in agreement with studies showing that increased NCX expression/activity was identified as a mechanism promoting heart failure, cardiac ischemia and arrhythmia [39,54,55,56]. For example, Pott et al. [57] demonstrated that NCX upregulation represents an independent proarrhythmic factor promoting early and delayed afterdepolarizations (EADs/DADs) in ventricular cardiomyocytes. Similarly, Bögeholz et al. [58] reported that the genetic NCX inhibition via heterozygous NCX-knockout (KO) protected against EADs in ventricular cardiomyocytes. Finally, the pharmacological inhibition of NCX with SEA0400 in a transgenic NCX-overexpressor mouse model suppressed the number of proarrhythmic spontaneous Ca^2+^ release events [59]. In summary, the arrhythmias caused by -AA+YWF, which augments the proteasome nuclear signal, are likely triggered by the enhanced activity of the NCX reverse mode, resulting in increased intracellular Ca^2+^.

### 3.4. The Association between Proteasome Translocation and the Arrhythmias

Our findings show that complete AA starvation causes proteasome translocation from the nucleus to the cytoplasm (i.e., decreased nuclear proteasome and increased cytoplasm proteasome), while the addition of YWF (-AA+YWF) blocks this nuclear export and increases the nuclear proteasome at the expense of the cytoplasm compartment. Concomitantly, both treatments cause arrhythmias, albeit of different properties: (i) at the single-cell level, the arrhythmias caused by -AA+YWF are more pronounced than by -AA. At the network level, arrhythmias were only caused by -AA+YWF, not by -AA. (ii) In contrast to -AA treatment, the arrhythmias caused by -AA+YWF exhibit bimodal firing patterns. Our findings present an apparent dilemma: while both treatments cause arrhythmias (although with different features), they induce opposite effects on proteasome compartmentalization. To account for this seeming discrepancy, we propose the following model. -AA treatment causes mild arrhythmias, possibly due to AA starvation per se, partially compensated by the proteasome translocation to the cytoplasm, to replenish the medium-depleted AAs. We further propose that supplementing the starvation medium (-AA) with YWF blocks/negates the coping mechanism mediated by the proteasome export to the cytoplasm, thus increasing the nuclear proteasome signal/activity, which either directly or indirectly augments the reverse mode of NCX activity. This in turn increases intracellular Ca^2+^, thereby triggering pronounced arrhythmias. This novel concept is supported by the finding that supplementing -AA with LMB (which blocks protein nuclear export) causes both increased proteasome nuclear localization as well as arrhythmias, similar to YWF.

### 3.5. Summary

This study presents novel findings regarding the interaction between nutrient deficient-induced proteasome compartmentalization, abnormal intracellular Ca^2+^ handling and arrhythmias. A major finding was that, in both the CMs (at the single-cell and network levels) and in retrogradely perfused isolated rat hearts, AA-deficient media cause arrhythmias. Collectively, our findings describe a newly identified mechanism linking the UPS to the arrhythmia generation in CMs and whole hearts.

## 4. Materials and Methods

### 4.1. iPSCs Culture and Differentiation

In the current study, we used the previously described human iPSC-CMs clones 24.5 [36] and FSE [60]. iPSC differentiation into cardiomyocytes was performed according to the previously described directed differentiation by modulating Wnt/β-catenin signaling protocol [61]. iPSCs were cultured on Matrigel (GFR, BD Biosciences, Franklin Lakes, NJ, USA)-coated 6-well plates in mTeSR1 medium (Stemcell Technologies, Vancouver, Canada) for 5–6 days. To initiate differentiation, cells were incubated with 1 mL/well Versene solution (Invitrogen, Life Technologies, Woburn, MA, USA) at 37 °C for 5 min and seeded on Matrigel-coated 12-well plates, in mTeSR1 medium. The medium was replaced daily, and, after 2 days when the monolayer of cells reached 100% confluence, the medium was changed to RPMI supplemented with B27 minus insulin (Invitrogen, Life Technologies, Woburn, MA, USA) containing 10 μM CHIR99021; this day was counted as day 1 of differentiation. On the next day (day 2 of differentiation), the medium was changed to RPMI supplemented with B27 minus insulin. On day 4, the medium was changed to RPMI supplemented with B27 minus insulin, containing 10 μM of IWP-4. On day 6, the medium was changed to RPMI supplemented with B27 minus insulin. Finally, from day 8 onwards, the medium was supplemented with RPMI with B27 complete supplement (Invitrogen, Life Technologies, Woburn, MA, USA). A total of no less than ten different differentiation protocols were utilized throughout this study.

### 4.2. Enrichment of Cardiomyocytes

To increase the fraction of cardiomyocytes in the culture, MACS magnetic labeling-based cell sorting kit was used (130-110-188, Miltenyi Biotec, Bergisch Gladbach, Germany). To generate beating monolayers, 25–50K enriched cardiomyocytes were seeded in a ~3 mm diameter circle on glass coverslips, formed by a 40 µL Matrigel droplet.

### 4.3. Langendorff Retrogradely Perfused Isolated Rat Hearts

The study was approved by the Animal Care and Use Committee of the Technion (IL-081-06-17) and performed using male Wister rats, 6–8 weeks old and weighing 175–200 g. The animals were sacrificed (IP urethane 1.6 mg/kg) and the hearts transferred to a custom-built chamber perfused using a Langendorff apparatus with oxygenized solution. The chamber was maintained at 37 °C throughout the experiments. Retrograde aortic perfusion was maintained for 1.5 hr. Two leads ECG tracings (equivalent to ECG leads I and II) were continuously recorded during the experiment. The time for the emergence of cardiac arrhythmias from the initiation of reperfusion was evaluated. ECG analysis was performed with Lab Chart Pro v.8.1.16 (AD Instruments, Sydney, Australia). Arrhythmias were defined as the presence of ventricular fibrillation (VF) or sustained ventricular tachycardia (VT) (e.g., lasting more than 30 s) within the 1.5 hr perfusion.

### 4.4. Immunofluorescence Staining

CMs were plated in glass-bottomed wells coated with Matrigel (GFR, BD Biosciences, Franklin Lakes, NJ, USA). Following the indicated treatments, cells were fixed with 4% paraformaldehyde as previously described [21]. Indirect immunofluorescent staining was performed using the following antibodies: anti-α6 (1:25; hybridoma-derived [62]), anti-α-actinin (1:250, Abcam, Cambridge, Cambridgeshire, UK), anti-mouse Alexa fluor 488 (1:1000, Invitrogen, Life Technologies, Woburn, MA, USA), and anti-rabbit Alexa fluor 568 (1:000, Invitrogen, Life Technologies, Woburn, MA, USA). 

### 4.5. Live Imaging of Proteasome Activity

CMs were plated in glass-bottomed wells, coated with Matrigel (GFR, BD Biosciences, Franklin Lakes, NJ, USA). Following the indicated treatments, the proteasome activity probe Me4BodipyFL-Ahx3Leu3VS (I-190, R&D Systems, Burlington, MN, USA) [22,63] was added to the culture medium to a final concentration of 1 μM, and nuclei were stained using Hoechst 33342 (5 μg/mL) per manufacturer protocol (B2261, Sigma-Aldrich, Burlington, MA, USA). Following incubation for 30 min at 37 °C, CMs were visualized using a fluorescent confocal microscope (LSM 700, Zeiss, Oberkochen, Germany). The imaging conditions were for the Hoechst channel: excitation wavelengths 535 nm; emission wavelengths 465 nm. The imaging conditions for the probe channel: excitation wavelengths 488 nm; emission wavelengths 509.

### 4.6. Image Analysis 

Two-dimensional image analysis and fluorescence density measurements were conducted using Fiji software version 1.54 ImageJ) [64]. To maintain consistency across all cells, a constant threshold level was set during the analysis. To calculate the mean proteasome signal in the nucleus and cytoplasm (i.e., the signal density), the raw density (RawintDen) values of the proteasome (α6) were divided by respective nucleus or cytoplasm areas. Notably, to determine the density ratio, the nucleus density in each cell was divided by the cytoplasmic density, providing information about the intensity of the proteasome signal.

### 4.7. Drugs and Chemicals

KB-R7943, a selective inhibitor of NCX reverse-mode (NCX, 182004-65-5, Sigma-Aldrich, Burlington, MA, USA); Leptomycin B (LMB), a selective inhibitor of Exportin1 (L2913, Sigma-Aldrich, Burlington, MA, USA); caffeine (Fluka Analytical, Honeywell, Charlotte, NC, USA).

### 4.8. Action Potential Recordings from Small iPSC-CMs Clusters

Small clusters of cardiomyocytes digested by 3 min incubation in Trypsin EDTA (0.25% and EDTA 0.05%) were placed over Matrigel-coated glass coverslips. Following plating, recovery period of at least two days was allowed before performing electrophysiological experiments. Action potentials were recorded from spontaneously contracting small cardiomyocytes clusters. The experiments were conducted in external Tyrode’s solution containing (in mM) 140 NaCl, 5.4 KCl, 1 MgCl_2_, 2 sodium pyruvate, 1 CaCl_2_, 10 HEPES, and 10 glucose (pH 7.4 adjusted with NaOH). Axopatch 200B, Digidata 1322, and pClamp10 (Molecular Devices, Sunnyvale, CA, USA) were used for data amplification, acquisition and analysis. Signals were digitized at 10 kHz and filtered at 2 kHz. Patch pipettes with resistances of 4–7 MΩ were pulled from borosilicate glass capillaries (Harvard Apparatus, Holliston, MA, USA). Analysis was preformed using MATLAB 2016b software (MathWorks, Natick, MA, USA). The patch pipette solution contained (mM): 120 KCl, 1 MgCl_2_, 3 Mg-ATP, 10 HEPES, and 10 EGTA titrated to pH 7.2 with KOH and adjusted at 290 mOsm with saccharose.

### 4.9. Recordings of Extracellular Electrograms from Small iPSC-CMs Clusters

The microelectrode array (MEA) setup (Multi Channels Systems, MCS, Reutlingen, Germany) consists of a 50 × 50-mm glass substrate, in the center of which is embedded a 1.4 × 1.4 mm matrix of 60 titanium-nitride electrodes. Each electrode diameter is 30 µm and inter-electrode distance is 200 µm. Extracellular electrogram recordings (at 10 kHz with 12-bit precision) from contracting CMs clusters were performed using a PC-based data acquisition system consisting of the MEA, preamplifiers, filter amplifier, data acquisition board, and software. An additional PC was connected using an SD64 channel splitter to allow measurements using the MC_Rack 4.6.2 software (Multi Channel Systems MCS GmbH., Reutlingen, Germany). The signal was sampled at 1000 Hz, downsampled to 200 Hz, and stored in a binary file for offline processing. The recordings were conducted in the presence of Tyrode’s solution, and the temperature within the MEA chamber was kept at 37 ± 0.1 °C by means of temperature-maintaining system (GEFRAN 800, Montreal, QC, Canada).

### 4.10. Measurements of Intracellular Ca^2+^ Transients

Intracellular Ca^2+^ transients were recorded from small contracting CMs clusters by means of fura-2 fluorescence, using the IonOptix Calcium and Contractility system (IonOptix, Westwood, MA, USA) [33,49,65]. In brief, spontaneously contracting clusters were mechanically dissected and adhered onto 18 mm diameter gelatin-coated glass slides. Subsequently, fura-2-stained (2.5 μM) contracting areas were transferred to a chamber mounted on the stage of an inverted microscope and perfused at a rate of 1–1.5 mL/min Tyrode’s solution at 37 °C. The clusters were paced at 0.5–2.5 Hz, which corresponded to a frequency 20–50% higher than the spontaneous beating rate. The acquisition rate of the Ca^2+^ transients was 100 points/s. Analysis was performed by averaging 20 consecutive signals using the IonOptix designated system. To characterize the Ca^2+^ transients, the following parameters were calculated: amplitude (R_Amp_), and the maximal rates of [Ca^2+^]_i_ rise (+d[Ca^2+^]_i_/dt) and decay (−d[Ca^2+^]_i_/dt).

### 4.11. Analysis of Beat Rate Variability (BRV)

BRV was calculated from action potential and extracellular electrograms, based on the inter-beat intervals (IBIs) versus time plots [24,25]. The recordings were analyzed for peak detection of all recorded signals, and IBIs were calculated using dedicated MATLAB 2016b software. BRV measures were calculated from the Poincaré plot, in which each IBI_(n+1)_ is plotted against its predecessor IBI_(n)_, creating a scattered mass of points in a two-dimensional array. Quantitative analysis of the plot was performed by fitting an ellipse to the group of points, with its center coinciding with the centroid of the ellipse (the point of the average IBI) and adjusting two perpendicular lines traversing the centroid. The longitudinal line, designated SD2, represents long-term variability of the data (reflecting the standard deviation of the IBIs), while the perpendicular line, designated SD1, represents short-term IBI variability.

### 4.12. The Composition of the Control, -AA and -AA+YWF Solutions

(i). Tyrode’s solution to which physiological amounts of all amino acids (AAs) were added, Control: L-Alanine 4.45 mg/L, L-Arginine 69.46 mg/L, L-Asparagine 6.6 mg/L, L-Aspartic acid 6.65 mg/L, L-Cysteine 48 mg/L, L-Glutamic acid 7.35 mg/L, L-Glutamine 292 mg/L, L-Glycine 30 mg/L, L-Histidine 31.09 mg/L, L-Isoleucine 105 mg/L, L-Leucine 105 mg/L, L-Lysine 116.86 mg/L, L-Methionine 30 mg/L, L-Phenylalanine 66 mg/L, L-Proline 17.25 mg/L, L-Serine 42 mg/L, L-Threonine 95 mg/L, L-Tryptophan 16 mg/L, L-Tyrosine 72 mg/L, and L-Valine 94 mg/L). pH levels were adjusted to 7.4 with NaOH.
(ii). -AA, amino acid-free Tyrode’s solution.
(iii). -AA+YWF: Tyrode’s solution containing L-Tyrosine 362.38 mg/L, L-Tryptophan 408.46 mg/L and L-Phenylalanine 330.4 mg/L.

### 4.13. Statistical Analysis

The statistical analysis was performed using Prism 10 (GraphPad Software, San Diego, CA, USA). The specific test is described in the legend of each figure. The results are presented as mean ± SD. 

## Data Availability

Data are contained within the article.
